# Short-term outcomes of community-based adolescent weight management: The Loozit^® ^Study

**DOI:** 10.1186/1471-2431-11-13

**Published:** 2011-02-08

**Authors:** Vanessa A Shrewsbury, Binh Nguyen, Janice O'Connor, Katharine S Steinbeck, Anthea Lee, Andrew J Hill, Smita Shah, Michael R Kohn, Siranda Torvaldsen, Louise A Baur

**Affiliations:** 1 University of Sydney Clinical School, The Children's Hospital at Westmead, Sydney, Australia; 2 University of Sydney, Sydney, Australia; 3 Academic Unit of Psychiatry & Behavioural Sciences, Institute of Health Sciences, Leeds University School of Medicine, Leeds, UK; 4 Primary Health Care Education and Research Unit, Sydney West Area Health Service, Sydney, Australia; 5 Department of Adolescent Medicine, The Children's Hospital at Westmead, Sydney, Australia; 6 School of Public Health and Community Medicine, University of New South Wales, Sydney, Australia

## Abstract

**Background:**

The Loozit^® ^Study is a randomised controlled trial investigating extended support in a 24 month community-based weight management program for overweight to moderately obese, but otherwise healthy, 13 to 16 year olds.

**Methods:**

This pre-post study examines the two month outcomes of the initial Loozit^® ^group intervention received by both study arms. Adolescents (n = 151; 48% male) and their parents separately attended seven weekly group sessions focused on lifestyle modification. At baseline and two months, adolescents' anthropometry, blood pressure, and fasted blood sample were assessed. Primary outcomes were two month changes in body mass index (BMI) z-score and waist-to-height-ratio (WHtR). Secondary outcomes included changes in metabolic profile, self-reported dietary intake/patterns, physical and sedentary activities, psychological characteristics and social status. Changes in outcome measures were assessed using paired samples t-tests for continuous variables or McNemar's test for dichotomous categorical variables.

**Results:**

Of the 151 adolescents who enrolled, 130 (86%) completed the two month program. Among these 130 adolescents (47% male), there was a statistically significant (P < 0.01) reduction in mean [95% CI] BMI (0.27 kg/m^2 ^[0.41, 0.13]), BMI z-score (0.05 [0.06, 0.03]), WHtR (0.02 [0.03, 0.01]), total cholesterol (0.14 mmol/L [0.24, 0.05]) and low-density lipoprotein cholesterol (0.12 mmol/L [0.21, 0.04]). There were improvements in all psychological measures, the majority of the dietary intake measures, and some physical activities (P < 0.05). Time spent watching TV and participating in non-screen sedentary activities decreased (P < 0.05).

**Conclusions:**

The Loozit^® ^program may be a promising option for stabilizing overweight and improving various metabolic factors, psychological functioning and lifestyle behaviors in overweight adolescents in a community setting.

**Trial registration:**

Australian New Zealand Clinical Trials Registry

ACTRNO12606000175572

## Background

Adolescent obesity is a significant public health issue [1] often associated with a range of medical [2-5] and psycho-social problems [6]. Family-based lifestyle interventions are the recommended first line of treatment for adolescent obesity [7] and have a modest capacity to reduce overweight [8] and improve metabolic risk factors [9]. Much of the research has focused on outcomes of intensive clinical programs offered at tertiary treatment centers [8]. Community-based adolescent group programs for obesity treatment are a relatively understudied intervention [10].

Potential advantages of community-based group management of adolescent obesity over treatment in the tertiary setting include greater accessibility for participants, fewer time constraints, and more interactive knowledge and skill building opportunities [10]. There is a pressing need for research to evaluate the clinical and psycho-social outcomes of lower intensity, and potentially economically sustainable, community-based lifestyle interventions for adolescent weight management.

Our pilot work, in Sydney, Australia, established that a program with such features offered through community health centers and involving community-based recruitment, was feasible and acceptable to adolescents. Importantly it was accompanied by a reduction in waist circumference and improvements in high density lipoprotein cholesterol and aspects of self-perception [11]. Participant feedback from the pilot prompted changes to the program such as the involvement of parents and more sessions over a shorter time span; it is now called the Loozit^® ^group program [12]. This study aimed to examine the short-term (2 month) anthropometric, metabolic, behavioral, and psycho-social outcomes of the Loozit^® ^group program.

## Methods

### Study design

This paper describes Phase 1 (2 month outcomes) of the Loozit^® ^two-arm randomized controlled trial (RCT) for weight management in overweight to moderately obese adolescents. The Phase 1 intervention is a low-moderate intensity (i.e. one contact per week) community-based group lifestyle program that is delivered identically to both study arms and therefore is evaluated as a pre-post study in the present paper. The full RCT protocol, including a detailed description of the Phase 1 intervention, has been published elsewhere [12]. Briefly, the Phase 2 intervention, which is still underway, involves participants in both study arms attending group sessions approximately once every three months from 2 months to the completion of the study at 24 months. One study arm also receives additional therapeutic contact in the form of telephone coaching, short-message service text messaging and/or email messages. This study is registered with the Australian New Zealand Clinical Trials Registry (ACTRNO12606000175572) and has been approved by the Human Research Ethics Committees of The Children's Hospital at Westmead, Sydney West Area Health Service, and The University of Sydney.

### Participant recruitment

Between May 2006 and May 2009, adolescents were recruited in Sydney, Australia, by community-based recruitment, primarily via schools, the media, health professionals and community organizations. Eligibility to participate in the study was initially assessed via a telephone screen and was confirmed at a face-to-face appointment. Adolescents were eligible to participate if they were: 13 to16 years old; overweight to moderately obese (i.e. body mass index (BMI) z-score range 1.0-2.5) but otherwise healthy; available to attend the scheduled Phase 1 group sessions with a parent/carer; able to access a landline telephone and a mobile phone or email (relevant to the Phase 2 intervention). A BMI z-score of 1.0 is equivalent to the 85^th ^percentile on the Centers for Disease Control and Prevention (CDC) BMI-for-age growth chart i.e. the lower boundary for defining overweight in children and adolescents. We excluded severely obese adolescents (i.e. BMI z-score >2.5) because they are more likely to have comorbid conditions and thus require more intensive and individualized help offered in tertiary treatment settings. Additional exclusion criteria were: a poor level of spoken English; an intellectual or physical disability; a secondary cause for the obesity; or taking medications that affect weight status. Informed consent to participate in this study was obtained in writing from adolescents and their parent/carer.

### Intervention

All adolescents in the study received the Loozit^® ^group program during Phase 1. The program involved seven × 75 minute group sessions held once per week in separate rooms for adolescents and their parents/carers. Trained dietitians facilitated the groups involving 5-9 participants held at a suburban community health center or in school rooms at a children's hospital. The particular settings were chosen because they were readily accessible to members of the community and were available free of charge to the study investigators. The program is based on the social cognitive theory to change dietary intake and activity levels, and to modify self-efficacy, motivation, perseverance and self-regulation [13]. The initial session focuses on the benefits of healthy living and encourages setting goals at least once per week throughout the program. The second session discusses increasing physical activity and reducing sedentary behaviors. The next two sessions focus on healthy eating. Adolescents' session five covers stress management, and session six focuses on building positive self esteem. The final session summarizes the previous sessions and discusses techniques for maintaining positive changes. All adolescent sessions include a total of 20 minutes of indoor resistance activities and fun active games. Parent sessions focus on practical support of behavioral change in adolescents and role modelling of healthy lifestyle behaviors. A detailed description of the content covered in each group session has been published elsewhere [12].

### Adolescent outcomes

#### Data collection procedures

Adolescents attended an initial appointment with a parent/carer to assess baseline anthropometry and pubertal stage, to complete demographic questionnaires, and to arrange fasting venipuncture at an external pathology laboratory. At the two month follow up anthropometry and instructions for the fasting venipuncture was repeated. Measuring equipment was regularly calibrated and the physical outcome assessors attended measurement training sessions. Adolescents attended a group session at baseline and two months to complete individual questionnaires on behavioral and psycho-social outcomes.

#### Anthropometry and metabolic indicators

Portable scales (Tanita HD-316, Tanita Corp., Tokyo, Japan) were used to measure weight to the nearest 0.1 kg, with shoes and heavy clothing removed. Height was measured to the nearest 0.1 cm using a fixed stadiometer (Holtain Limited, Wales, UK) at the children's hospital or a portable stadiometer (Seca, Model 220, Hamburg, Germany) at the community health center. Waist circumference (WC) was measured at the narrowest point between the lower costal (rib) border and the iliac crest using a nonextensible steel tape. The primary outcomes were BMI z-score, based upon age-and sex-specific reference values [14], and the waist-to-height ratio (WHtR). Since the development of the Loozit^® ^Study protocol in 2005 (and later published [12]), WHtR has been established as a simple, age-independent, measure of abdominal adiposity and cardiovascular risk factor clustering [15,16] and hence has been reported instead of waist circumference z-score. Systolic and diastolic blood pressure (BP) were measured using an automated BP monitor (Dinamap model 8101, Critikon Inc., FL) under standard conditions [17]. A nationally accredited pathology laboratory collected fasting blood samples and assessed: total cholesterol including high density (HDL) and low density lipoprotein (LDL) fractions, triglycerides, insulin, glucose and alanine aminotransferase (ALT). The homeostasis model assessment of insulin resistance (HOMA-IR) was calculated ([fasting insulin (mU/L) × fasting glucose (mmol/L)]/22.5) [18]. Participants were reimbursed AUD $20.00 for travel expenses associated with blood collection.

#### Lifestyle behaviors

Physical activity and sedentary behavior were assessed using the validated Children's Leisure Activities Study Survey [19]. Time spent in total physical activity (the sum of 42 activities) and at various intensity levels (light, moderate, and vigorous [20,21]) was calculated. Sedentary leisure activities were classified as screen based and non-screen based. Participants whose sedentary leisure activity time exceeded 72 hours/week were excluded according to established protocols [22]. Adolescents' adherence to national guidelines [23] recommending daily participation in at least one hour of moderate to vigorous physical activity and no more than two hours/day of screen pursuits was assessed. Dietary intake was measured using a food frequency questionnaire [24] with additional questions on eating behaviors that were used in an Australian study of adolescent dietary intake [25]. Responses were categorised into dichotomous variables to indicate whether or not adolescents met Australian dietary recommendations [26].

#### Psycho-social factors

The Mental Health Inventory-5 (MHI-5) score (5 = most favorable health; 30 = least favorable health), based on a five-question mental health assessment component of the SF-36, was used to assess quality of life [27]. Sex specific, 9-figure scales ranging from thin to fat body shapes (scoring: 1 to 9) investigated body shape perception. Participants made two choices: current perceived body shape and ideal body shape with body dissatisfaction being the difference between the two [28]. The MacArthur Scale of Subjective Social Status, an adaptation of a 10-point vertical ladder scale (1 = extremely low; 10 = extremely high), was used to evaluate perceived social acceptance with adolescent peers [29]. The 45-item Self Perception Profile for Adolescents was used to assess perceived mean competence in eight domains (scholastic, social acceptance, athletic, physical appearance, job, romantic appeal, close friendship, and behavioral conduct) as well as global self-worth (scoring: 1 = low; 4 = high) [30]. This tool includes an additional 16-item measure to assess the level of importance that adolescents attribute to each domain.

### Baseline variables

#### Pubertal stage

Adolescents self-reported their stage of pubertal maturation using the standard Tanner Stage line drawings and menarchal status for females [31]. Early puberty was defined as Tanner Stages 1-2 for male genitalia and pre-menarche in females. Mid/late puberty was defined as Tanner Stages 3-5 for male genitalia and post-menarche in females.

#### Demographic characteristics

A parent/carer completed a questionnaire including the following items: maternal and paternal highest education level and birthplace; residential postal area code; and primary language spoken at home. Parental birthplace was classified using the Australian Standard Classification of Cultural and Ethnic Groups [32]. The Australian Bureau of Statistics 2006 Socio-Economic Indexes for Areas (SEIFA) Index of Relative Socio-economic Advantage and Disadvantage (IRSAD) was assigned to each residential postal area code. IRSAD is a general index that includes 21 measures and represents a continuum of advantage (high values) to disadvantage (low values) [33].

### Participant program evaluation

At the two month follow up adolescents and parents completed an anonymous evaluation questionnaire, adapted from a study involving obese pre-adolescent children [34]. Using Likert scales, participants assessed various aspects of the Loozit^® ^group program including quality, usefulness of the content/resources, and overall satisfaction. Participants were asked if they would recommend the program to other people.

### Statistical analysis

#### Sample size

It was estimated that a sample size of 128 (i.e. 64 per intervention arm) would provide 80% power to detect a 0.4 unit difference in mean change of BMI z-score from baseline to 2, 12 and 24 months follow up in the two arms in the forthcoming RCT (two group t-test, 0.05 two-tailed significance).

#### Baseline to two month changes

Data entry was checked by a second researcher and analyzed using SPSS 17.0 (SPSS Inc., Chicago, IL). Of the enrolled adolescents (n = 151), dropouts are defined as those who withdrew from the study prior to the first group session (n = 14) or during the intervention (n = 7). Two month changes in anthropometry, metabolic and psycho-social outcomes in adolescents who completed the program were assessed using paired samples t-tests for continuous variables or McNemar's test for dichotomous categorical variables.

## Results

### Participant baseline characteristics

**Figure 1:**
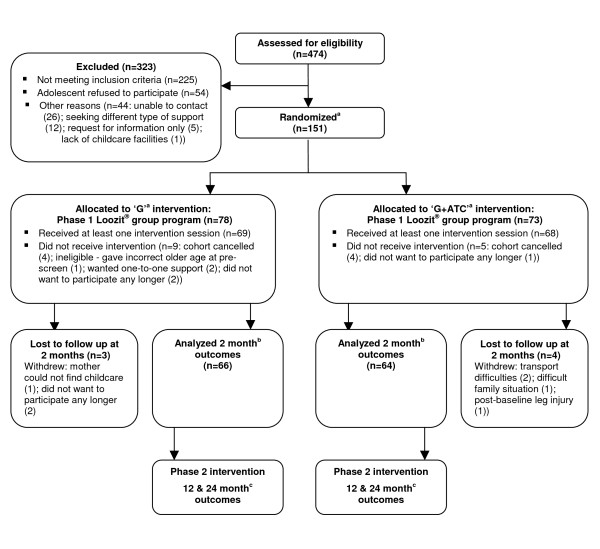
**Participant flow in the Loozit^® ^Study**. **Footnote**: _a _Abbreviations: G - group only intervention; G + ATC - group + additional therapeutic contact intervention involving telephone coaching and SMS/email communication. ATC commences after 2 month outcome assessment. _b _Only 2 month outcomes are reported in this paper. Both study arms have received the same intervention thus far and therefore are analysed as one group. _c _Data collection is underway for 12 & 24 month outcomes and is expected to be completed in 2011. Differences between study arms will be reported.

Participant flow in the study is shown in Figure 1. From 474 enquiries, 323 adolescents were considered ineligible to participate in the study. The main reasons for ineligibility were adolescents being too young (below 13 years), difficulties accessing the venue (timing, location, transport problems, or lack of childcare facilities) and adolescents refusing to attend the program. Demographic characteristics of the 151 adolescents enrolled in the study and their parents are shown in Table 1. Mid/late stage of puberty was identified in 86% of females and 64% of males. Families with a university educated mother were less likely to complete the study (odds ratio 0.27 [95% CI: 0.10 to 0.72]) than those with a non-university educated mother. Adolescents who completed the program (n = 130; female 53%) and those who dropped out were not different in terms of other baseline demographic or anthropometric characteristics.

**Table 1:** Baseline demographic characteristics of adolescents and their parents

Characteristics (n = 151)	
*Adolescent*	
Median (interquartile range) age in years^a^	13.9 (13.4,14.8)
Female (%)	52
Mean (SD) SEIFA^a, b^	1054 (84)
Primary language spoken at home (%)^c^	
English	68
Arabic	7
Tagalog	5
Other^d^	20
	
*Parental*	
Dual parent households (%)	75
Region of birth^c ^- Mother (%): Father (%)	
Australia	59:49
South-East Asia	8:10
North Africa and Middle East	7:10
Southern-Central Asia	7:5
North-West Europe	5:7
Oceania	4:7
Other^e^	10:12
University degree (%):	
Mothers	38
Fathers	31

^a ^Range: Age in years:12.9 to 16.8; SEIFA: 865 to 1202

^b ^Socioeconomic Index for Areas Index of Relative Socioeconomic Advantage and Disadvantage. Mean for the Sydney Major Statistical Region is 1053

^c ^Based on the Australian Standard Classification of Cultural and Ethnic Groups

^d ^This group is comprised of 24 different primary languages spoken at home by three or fewer participants.

^e ^Less than 5% of mothers and fathers were born in North or South America, Southern Europe, South-East Europe, North-East Asia, or Sub-Saharan Africa.

### Changes in outcome measures between baseline and two month follow up

#### Anthropometry and metabolic indicators

Among adolescents who completed the program, there were statistically significant mean reductions in BMI, BMI z-score, WC and WHtR (Table 2). At two months, 22% had reduced BMI z-score by more than five percent and 38% had reduced WHtR by more than five percent. Total cholesterol and LDL cholesterol significantly decreased in adolescents who completed their two month blood test.

**Table 2:** Change in anthropometry and metabolic indicators between baseline and two months

	n^a^	Baseline	2 month	Δ Mean	P value^b^
		Mean (SD)	Mean (SD)	(95% CI)	
**Weight (kg)**	129	83.4 (14.6)	83.2 (14.7)	-0.19 (-0.58, 0.18)	0.336
**BMI (kg/m^2^)**	129	30.9 (3.9)	30.6 (4.0)	-0.27 (-0.41, -0.13)	0.0002
**BMI z-score**	129	2.03 (0.31)	1.99 (0.34)	-0.05 (-0.06, -0.03)	<0.0001
**WC (cm)**	129	97.0 (10.6)	94.6 (10.2)	-2.34 (-3.87, -0.81)	0.003
**Waist-to-height ratio**	129	0.59 (0.06)	0.58 (0.05)	-0.02 (-0.03, -0.01)	0.001
**Systolic BP (mm/Hg)**	129	119 (13)	120 (12)	1 (-1, 3)	0.272
**Diastolic BP (mm/Hg)**	129	60 (9)	60 (9)	0 (-2, 2)	0.959
**Triglycerides (mmol/L)**	102	1.4 (0.9)	1.3 (0.9)	0.00 (-0.13, 0.12)	0.949
**Total cholesterol (mmol/L)**	102	4.4 (0.8)	4.3 (0.8)	-0.14 (-0.24, -0.05)	0.003
**LDL cholesterol (mmol/L)**	101	2.5 (0.7)	2.4 (0.6)	-0.12 (-0.21, -0.04)	0.006
**HDL cholesterol (mmol/L)**	102	1.3 (0.3)	1.2 (0.3)	-0.04 (-0.08, 0.01)	0.085
**Glucose (mmol/L)**	102	4.8 (0.5)	4.7 (0.5)	-0.08 (-0.18, 0.02)	0.133
**Insulin (mU/L)**	102	20.0 (9.9)	19.2 (9.9)	-0.83 (-2.70, 1.03)	0.377
**HOMA-IR**	102	4.3 (2.4)	4.1 (2.3)	-0.25 (-0.69, 0.20)	0.276
**ALT (U/L)**^c^	102	24.3 (20.6)	22.8 (13.8)	-1.59 (-4.53, 1.35)	0.287

Abbreviations: CI, Confidence Interval; BMI, Body Mass Index; WC, Waist Circumference; BP, Blood Pressure; HDL, High Density Lipoprotein; LDL, Low Density Lipoprotein; HOMA-IR, Homeostasis Model Assessment of Insulin Resistance; ALT, Alanine Aminotransferase

^a ^Of the 130 adolescents who completed the 2 month program, one adolescent with self-reported anthropometry was excluded from the data analysis leaving 129 cases. With regards to the blood data, 20 study completers refused to have a blood test at either time point and a further 8 study completers who had a blood test in the non-fasted state were also excluded (one adolescent also had invalid LDL data).

^b ^Paired samples t-test

^c ^As the baseline and 2 month data were not normally distributed, this variable was log transformed but this did not have a substantial impact on the presented results

#### Behavioral measures

Reported changes in dietary intake, physical activity and sedentary behavior in adolescents who completed the program are shown in Table 3. Compared with baseline, there was a statistically significant improvement in the proportion of adolescents at two months whose reported intakes met dietary recommendations for fruit, vegetable, water, and breakfast consumption. This was accompanied by a statistically significant reduction in the reported frequency of consuming less desirable foods including high fat meat products, potato crisps, and sugary drinks. Compared with baseline levels, at two months adolescents reported spending significantly less time on screen based and non-screen based sedentary leisure activities. However, there was no change in reported time spent in total or specific intensities of physical activity, nor the proportion of adolescents reporting to meet guidelines for physical activity or screen time. At two months, adolescents reported spending more time in weight training (P < 0.001), walking the dog (P = 0.04) and dancing (P = 0.008) but there was no change in other listed activities.

**Table 3:** Reported dietary, physical activity and sedentary behavior changes between baseline and two months

Intake/Behaviors (Frequency)	n^a^	Baseline (%)	2 months (%)	Baseline to 2 months Δ in behavior/intake Reduced (%): No change (%): Increased (%)	P value
**Core food intake**					
Vegetables (≥ 4 serves/day)^b^	123	26	38	15:41:44	0.040^d^
Fruit (≥ 2 serves/day)^b^	119	71	83	18:54:28	<0.007^d^
**Extra food intake**					
High fat meat products (once/week or less)^c^	124	32	51	48:34:18	0.001^d^
Potato crisps (never or rarely)^c^	122	13	34	53:34:13	<0.001^d^
Fast food/takeaway (never or rarely)^c^	126	33	40	30:57:13	0.185^d^
**Drink intake**					
Water (≥ 6 cups/day)^b^	123	24	38	15:45:40	0.009^d^
Diet drinks (never or rarely)^c^	117	60	50	13:63:24	0.058^d^
Fruit juice/drink (never or rarely)^c^	120	28	43	36:48:16	0.002^d^
Regular sweetened drinks (never or rarely)^c^	120	46	63	31:55:14	0.001^d^
**Dietary behavior patterns**					
Consumes breakfast (everyday)^b^	128	52	61	8:62:30	0.035^d^
Consumes lunch (everyday)^b^	128	70	66	16:69:15	0.458^d^
Consumes dinner (everyday)^b^	128	82	87	5:82:13	0.238^d^
Makes or helps make dinner (≥ once/week)^b^	128	63	68	26:44:30	0.265^d^
Consumes dinner with most of family (everyday)^b^	129	57	52	20:64:16	0.281^d^
Dinner in front of TV (< once/week)^c^	129	47	50	27:57:16	0.541^d^
**Activities**					
Moderate-vigorous physical activity (> 1 hour/day)^b^	129	50	53	14:69:17	0.636^d^
Screen based leisurely pursuits (≥ 2 hours/day)^c^	82	28	32	15:74:11	0.664^d^

		**Baseline** **Mean (SD)**	**2 month** **Mean (SD)**	**ΔMean** **(SD)**	

**Total physical activity (hours/week)**	129	14.9 (8.7)	16.1 (11.5)	1.2 (11.1)	0.216^e^
Vigorous intensity	129	4.4 (4.1)	4.6 (5.5)	0.2 (5.0)	0.639^e^
Moderate intensity	129	4.5 (4.5)	5.0 (5.3)	0.4 (5.7)	0.391^e^
Light intensity	129	4.2 (3.3)	4.7 (3.7)	0.5 (3.7)	0.133^e^
**Total sedentary leisure activity (hours/week)**^f^	82	39.7 (16.0)	34.0 (15.7)	-5.7 (17.3)	0.004^e^
Screen based leisure pursuits	82	22.4 (11.1)	19.9 (11.0)	-2.5 (11)	0.04^e^
Watching TV/videos/DVDs	82	14.0 (8.0)	11.9 (7.7)	-2.1 (8.0)	0.02^e^
Using the computer/internet^g^	82	4.9 (5.2)	4.8 (6.3)	-0.2 (6.8)	0.817^e^
Playing electronic games	82	3.5 (5.5)	3.2 (5.1)	-0.3 (4.8)	0.580^e^
Non-screen based leisure pursuits	82	17.3 (11.1)	14.1 (11.5)	-3.2 (10.8)	0.009^e^

Abbreviations: SD - Standard Deviation

^a ^Data are reported for all adolescents who completed questionnaire items

^b ^The balance of adolescents consumed the food or performed the behaviour less often

^c ^The balance of adolescents consumed the food or performed the behaviour more often

^d ^McNemar's test

^e ^Paired samples t-test

^f ^48 adolescents who reported levels of sedentary leisure activities considered implausible (i.e. exceeding 72 hours/week) were excluded from these analyses as per established protocols [22]

^g ^Does not include school/homework

#### Psycho-social factors

At two months, there was a statistically significant improvement in the MHI-5 score, body shape dissatisfaction, global self-worth and most other domains of the Self Perception Profile (Table 4). The importance that adolescents placed on self-perception domains decreased for close friendship (P = 0.002) but did not change for any of the other domains.

**Table 4:** Change in psycho-social factors between baseline and two months

Domain	n^a^	Baseline	2 month	Δ Mean	Δ P value^b^
		mean (SD)	mean (SD)	(SD)	
**Mental Health Inventory (MHI-5)** **score^c^**	129	13.2 (4.6)	12.2 (4.2)	-1.0 (3.5)	0.002
**Body shape dissatisfaction^d^**	125	2.5 (1.0)	2.1 (0.9)	-0.4 (0.9)	<0.001
**Self-Perception Profile**	129				
Global self worth		2.59 (0.69)	2.76 (0.60)	0.17 (0.48)	<0.001
Scholastic competence		2.71 (0.73)	2.84 (0.68)	0.13 (0.51)	0.005
Social acceptance		2.94 (0.77)	3.03 (0.70)	0.09 (0.48)	0.035
Athletic competence		2.27 (0.74)	2.37 (0.74)	0.10 (0.49)	0.023
Physical appearance		1.86 (0.62)	2.09 (0.65)	0.23 (0.52)	<0.001
Job competence		2.99 (0.58)	3.12 (0.57)	0.12 (0.52)	0.007
Romantic appeal		2.34 (0.62)	2.47 (0.63)	0.12 (0.53)	0.009
Behavioral conduct		2.85 (0.71)	2.95 (0.65)	0.09 (0.46)	0.023
Close friendship		3.23 (0.80)	3.33 (0.66)	0.10 (0.63)	0.060
**Subjective social status^e^**	130	6.4 (2.0)	6.6 (2.0)	0.2 (1.8)	0.212

^a ^Data are reported for all adolescents who completed questionnaire items

^b ^Paired samples t-test

^c ^Scale: 5 = most favorable health; 30 = least favorable health

^d ^Scale: scores closer to zero indicate lower levels of body shape dissatisfaction

^e ^Scale: 1 = extremely low; 10 = extremely high

### Group session attendance & satisfaction

Attendance rates at group sessions progressively declined from week 1 to 7, ranging from 93% to 81% in adolescents and 93% to 74% in parents. Overall, adolescents' and parents' ratings indicated that they were highly satisfied with the program with 94% of adolescents and 100% of parents responding that they would recommend the program to others.

## Discussion

In this two month community-based group lifestyle intervention there was a stabilization in BMI and waist circumference in the majority of adolescent participants. A five percent or greater reduction in BMI z-score and WHtR was achieved by almost a quarter and over a third of adolescents respectively. These changes were accompanied by improvements in total and LDL cholesterol, psychological functioning, and self-reported lifestyle behaviors. The high attendance rates and satisfaction ratings indicate that the intervention was well received by adolescents and their parents.

There are several published studies of low to moderate intensity group lifestyle interventions in overweight adolescents [35-37] that are similar enough to compare to this study. Those studies, all from the USA, were published over twenty years ago and had less than half the sample size. The 14 week Shapedown RCT resulted in a reduced relative weight (actual weight divided by expected weight) at three months that was sustained at 15 months follow up when compared to a non-treatment control arm [35]; BMI was not reported. Two other RCTs examined the effect of a 16 week group treatment in three study arms i.e. adolescents with their mother (sessions together or separately) or alone in African-American females [36] and white lower-middle class families [37]. At the 16 week follow-up BMI decreased in both studies in all study arms (~1.3 kg/m^2 ^[36]; 3.3 kg/m^2 ^[37]) but tended to be less pronounced when the adolescent was treated alone. By six month [36] or 12 month [37] follow up participants had largely returned to their baseline BMI except in the separate mother-child arm in one study [37] where participants had maintained a reduced BMI.

A recent community-based RCT in 8 to 14 year olds, of similar intensity to our study in the first eight weeks (of a 16 week intervention), showed a BMI z-score reduction in children in the parent-only intervention compared with the control condition at both 4 month (0.127) and 10 month (0.115) follow up. A decrease in BMI z-score (0.136) was observed in children in the family-based intervention at 10 months only [38]. Three other interventions can be considered comparable with the Loozit^® ^group program, albeit with a greater intensity of contact [39-41]. These studies involved contact at least twice a week for three to six months with statistically significant reductions in mean BMI (0.16 kg/m^2 ^[39]; 2.1 kg/m^2 ^[40]) or BMI z-score (0.07 [41]) at 6 months.

It is evident from the present and comparative studies that in the short term (i.e. < 6 months) modest reductions in the level of overweight can be achieved although the magnitude of change may be related to the intervention intensity and duration. However, longer term outcomes in such interventions are rarely reported. The Loozit^® ^Study is designed to address this short-coming as the affect of additional therapeutic contact will be determined in a randomized trial with outcomes assessed at 12 and 24 month follow-ups [12].

This study, consistent with previous studies [36,37,40], has shown a modest reduction in various metabolic parameters, however the long term significance of these outcomes is unknown. The improvement in psychological functioning in the present study is consistent with the comparison studies that also examined depression and self-esteem [35,36]. These findings support the contention that lifestyle interventions do not have a detrimental impact on adolescent well-being in the short-term.

Of the previously mentioned comparison studies, one [35] examined changes in self-reported weight-related behaviors with improvements in overall 'behavior' observed at 3 and 15 months follow up. While reported intake of most dietary factors improved in the present study breakfast consumption was the only dietary behavior pattern to improve. It is apparent that improving dietary behavior patterns, in particular the frequency of eating together as a family away from the television, may need greater emphasis. The reported reduction in sedentary activities is encouraging although the deficit did not result in greater overall physical activity. The increase in weight training could be the result of each group session dedicating time to performing resistance activities and encouragement given to continue these exercises at home. Barriers to increasing overall physical activity were not specifically assessed; however, anecdotal feedback to group facilitators indicated that parents found it difficult to find activities that their adolescent enjoyed and to motivate them to be active.

A methodological limitation of this initial phase of the Loozit^® ^study was the absence of a control group. In designing this study, which has an active control group in Phase 2 (see Figure 1), we considered it unethical to have a non-treatment control group given that most RCTs of pediatric obesity lifestyle interventions show that such interventions are superior to control conditions [8]. Hence it is probable that the positive changes observed in this study are attributable to the intervention but this cannot be stated definitively. Another limitation of this study was that behaviors were self-reported. Nonetheless, even if the improvements in behaviors did not reflect reality, it does indicate an improvement in adolescents' knowledge of healthy lifestyles.

Participant recruitment was the most challenging aspect of conducting this study and an analysis of the efficacy and cost-effectiveness of various recruitment strategies has been reported elsewhere [42]. The demands of working with adolescents cannot be underestimated. The group facilitators worked hard to ensure an optimal balance between having fun (a retention strategy) and covering the session content in a timely manner. Multiple reminders to families were required to achieve pathology collection.

## Conclusions

Overall, a stabilization in the level of adolescent overweight was accompanied by improvements in several other outcomes. The Loozit^® ^program may be a promising resource for improving the health and well-being of overweight adolescents in a community setting. It is recommended that future research investigate techniques for improving the magnitude of overweight reduction in low-moderate intensity interventions such as the Loozit^® ^program. Future follow up of these adolescents at 12 and 24 months post-baseline will determine the extent to which low intensity extended support, delivered from 2 to 24 months post-baseline, further impacts on weight status and secondary outcome measures in this community-based weight management intervention.

## Competing interests

The authors declare that they have no competing interests.

## Authors' contributions

The study chief investigators JO, LAB, KSS, AJH, MRK and SS were responsible for identifying the research question, design of the study, obtaining ethics approval, the acquisition of funding, and overseeing study implementation. JO, AL, VAS, BN, KSS, LAB, and AJH contributed to developing the precise content of the study interventions and resources, and/or recruiting participants, and/or study implementation. JO, LAB, KSS, ST and VAS developed a detailed analysis plan for the study. VAS conducted the statistical analysis of all data with the exception of the physical activity and sedentary behavior outcomes which were analyzed by BN. All authors were responsible for the drafting of this manuscript and have read and approved the final version.

## Pre-publication history

The pre-publication history for this paper can be accessed here:

http://www.biomedcentral.com/1471-2431/11/13/prepub
